# The Use of Mobile Educational Tools to Improve Antimicrobial Prescription for the Treatment of Acute Pyelonephritis in Pregnancy: A Retrospective Cross-sectional Study

**DOI:** 10.1055/s-0039-1678590

**Published:** 2019-02

**Authors:** Vicente Sperb Antonello, Marjane Caroline Cunegatto, Elisa Tasca Rosin, Júlia Xavier Castilhos, Fabrícia Gamba Beduschi, Daniela Benzano Bumaguin, Ivan Carlos Ferreira Antonello, Mirela Foresti Jimenez

**Affiliations:** 1 Department of Prevention and Infection Control, Hospital Fêmina, Porto Alegre, RS, Brazil; 2 Graduate Program in Medicine and Health Sciences, Universidade do Vale do Rio dos Sinos (UNISINOS), São Leopoldo, RS, Brazil; 3 Department of Gynecology and Obstetrics, Hospital Fêmina, Porto Alegre, RS, Brazil; 4 Statistics Advisory Center, Universidade Federal do Rio Grande do Sul (UFRGS), Porto Alegre, Brazil; 5 Graduate Program in Medicine and Health Sciences, Pontifícia Universidade Católica do Rio Grande do Sul (PUCRS), Porto Alegre, RS, Brazil

**Keywords:** urinary tract infections, pregnancy, medical education, medical informatics application, infecções do trato urinário, gestação, educação médica, aplicações de informática médica

## Abstract

**Objective** To analyze the prescription of antimicrobial agents for pregnant women admitted into the obstetrics service who presented with acute pyelonephritis.

**Methods** Three cross-sectional studies were performed comparing the prescription of antimicrobials for pyelonephritis in pregnant women in the time periods evaluated (2010–2011: 99 patients evaluated; 2013: 116 patients evaluated; 2015: 107 patients evaluated), at the Hospital Fêmina, Porto Alegre, in the state of Rio Grande do Sul, Brazil. The analysis was performed before and after the promotion of an institutional protocol for the treatment of pyelonephritis during pregnancy, and on a third occasion after the introduction of a smartphone-based mobile educational tool.

**Results** The evaluation of the prescribing physicians and the adequacy of the prescriptions between the different periods studied revealed a significant increase in appropriate conduct for the choice of antimicrobial (2010: 83.8%; 2013: 95.7%; and 2015: 100%), route of administration (2010: 97%; 2013: 100%; and 2015: 100%), and interval (2010: 91.9%; 2013: 95.7%; and 2015: 100%), following the introduction of the protocol, and again after the implementation of the software application with orientations on the antimicrobial treatment.

**Conclusion** The use of specific mobile applications should be encouraged to attain a better quality and accuracy in prescriptions and to include strategies that not only reduce the risk of negative outcomes, but also improve the quality of care and treatment for maintaining the health both of the mother and of the baby.

## Abstract (pt)

**Resumo:**

**Objetivo** Analisar a prescrição de antimicrobianos para gestantes admitidas no serviço de obstetrícia que apresentaram pielonefrite aguda.

**Métodos** Foram realizados três estudos transversais comparando a prescrição de antimicrobianos para pielonefrite em gestantes nos períodos avaliados (2010–2011: 99 indivíduos avaliados; 2013: 116 indivíduos avaliados; 2015: 107 indivíduos avaliados), no Hospital Fêmina, Porto Alegre, RS, Brasil. A análise foi realizada antes e após a promoção de um protocolo institucional para o tratamento da pielonefrite durante a gravidez e, em uma terceira ocasião, após a introdução de uma ferramenta educacional móvel para uso por smartphone.

**Resultados** A avaliação das prescrições médicas e a adequação das prescrições entre os diferentes períodos estudados revelaram um aumento significativo na conduta adequada para a escolha do antimicrobiano (2010: 83,8%; 2013: 95,7%; e 2015: 100%), via de administração (2010: 97%; 2013: 100%; e 2015: 100%) e intervalo (2010: 91,9%; 2013: 95,7%; e 2015: 100%), após a introdução do protocolo, e novamente após a implementação do aplicativo com orientações sobre tratamento antimicrobiano.

**Conclusão** O uso de aplicativos móveis específicos deve ser incentivado para obter melhor qualidade e precisão nas prescrições e incluir estratégias que não apenas reduzam o risco de resultados negativos, mas que também melhorem a qualidade dos cuidados e do tratamento para manter a saúde conjunta da mãe e do bebê.

## Introduction

The current scenario, driven by technological innovations, has enabled health professionals to have immediate access to up-to-date information that supports medical decision-making.^1^ ^2^ ^3^ Mobile applications are frequently used in daily clinical practice (such as, Medscape, Epocrates and UpToDate). However, the information is often extensive and lacking objectivity, and its applicability in different locations is debatable.^1^ ^4^ This situation is particularly noticeable when considering the choice of antimicrobials for the treatment of infectious diseases.

Treatment protocols for urinary tract infection during pregnancy are scarce in the medical literature, and recommendations can vary regionally according to the local microbiota, with different antimicrobial resistance patterns.^5^ ^6^ Therefore, knowledge and ready access to up-to-date information on this subject are fundamental for the diagnosis and treatment to prevent maternal-fetal complications, such as maternal sepsis, premature birth, and low birth weight.^5^ ^7^ ^8^

The present study aimed to analyze the prescription of antimicrobial agents for pregnant women admitted to the Obstetrics Service of a reference hospital in the care of women in southern Brazil who presented a clinical case compatible with acute pyelonephritis. This analysis was performed before and after the promotion of an institutional protocol for the treatment of pyelonephritis during pregnancy, and on a third occasion after the introduction of a mobile educational tool for use with a smartphone, which provided guidelines for the diagnosis and treatment of pyelonephritis in pregnancy.

## Methods

A retrospective study was conducted to evaluate antimicrobial prescriptions given on admission to pregnant patients diagnosed with acute pyelonephritis. The evaluation took place over 3 different periods (2010, 2013 and 2015) at the Hospital Fêmina, in Porto Alegre, in the state of Rio Grande do Sul, Brazil. The hospital has an obstetric center, a surgical block, an adult intensive care unit (ICU), and a neonatal ICU, with 180 available beds. Hospital procedure requires the review of all antimicrobial prescriptions by an infectious disease physician from the Infection Control Service of the Hospital Fêmina.

For the purposes of the present research, the prescriptions given to pregnant women diagnosed with pyelonephritis were evaluated at 3 different points in time: 1) before the implementation of the institutional protocol for the diagnosis and treatment of pyelonephritis during pregnancy—data collected from June 2010 to December 2010; 2) after the implementation and dissemination of the institutional protocol for the diagnosis and treatment of pyelonephritis during pregnancy, which occurred in January 2011—data collected from January 2013 to December 2013; 3) after the implementation and dissemination of a smartphone application with objective and summarized data for the treatment of pyelonephritis during pregnancy, which occurred in December 2014—data collected from January 2015 to November 2015.

The diagnosis and treatment protocol for pyelonephritis during pregnancy was produced by the Hospital Infection Control Service and the Obstetrics Service of the Hospital Fêmina in January 2011. It was distributed via email to all doctors over the course of 2011 and 2012, and was made available on the prescription system to enable a quick and easy access. The protocol contains microbiology data for patients admitted to the hospital in 2010 and recommendations regarding diagnosis and antimicrobial therapy for pyelonephritis during pregnancy, with the first treatment choice being the intravenous (IV) administration of cefuroxime (0.75–1.5 g every 8 hours). The IV administration of a combination of amoxicillin (1 g) with clavulanate (0.2 g) every 8 hours, is suggested as an alternative. The same protocol recommends avoiding the use of oral β-lactams due to their ineffectiveness, as well as of drugs with a low susceptibility profile in isolated urine culture exams, such as ampicillin, cephalothin, and sulfamethoxazole/trimethoprim.^9^ Due to inadequate tissue levels, nitrofurantoin and fosfomycin are considered inappropriate for the treatment of pyelonephritis.

**Fig. 1:**
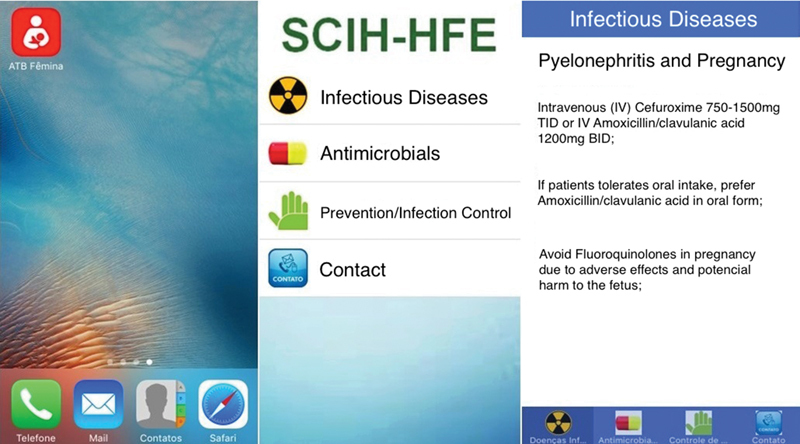
Layout of *ATB Femina mobile application*.

The ATB Fêmina smartphone application, available for iOS and Android platforms (Fig. 1), is a Portuguese language app, developed by the Hospital Infection Control Service of the Fêmina Hospital to facilitate quick access to objective data, with information regarding the diagnosis of infectious diseases during pregnancy and the appropriate specific treatment. The application was widely circulated to all the prescribing health professionals at the Hospital Fêmina, becoming extensively used by all. The information was based on the current literature and local microbiology, with the same recommendations for the treatment of pyelonephritis in pregnant women as was established by the protocol in 2011.^5^ ^6^ ^7^ ^8^

The authors compared the prescriptions issued by physicians upon the admission of pregnant women with pyelonephritis for each of the three time periods, in relation to the following variables: type of antimicrobial prescribed; dose; interval; and route of administration. A fifth variable was considered in accordance with the four aforementioned items, meaning that if all protocol steps were properly followed, the prescription was considered appropriate. This was designated as being *completely appropriate*. The cases in which there were one or more inconsistencies in the prescription were considered as inappropriate.

The categorical variables for the statistical analysis were described by frequencies and percentages. The chi-squared test was used to compare the frequency of satisfactory prescriptions. The analysis was performed with IBM SPSS Statistics for Windows, Version 20.0 (IBM Corp., Armonk, NY, USA), considering a significance level of 5%.

The present study was approved by the Research Ethics Committee of the Grupo Hospitalar Conceição on November 27th, 2015, under the registration number 50047715.9.0000.5530.

## Results

**Fig. 2:**
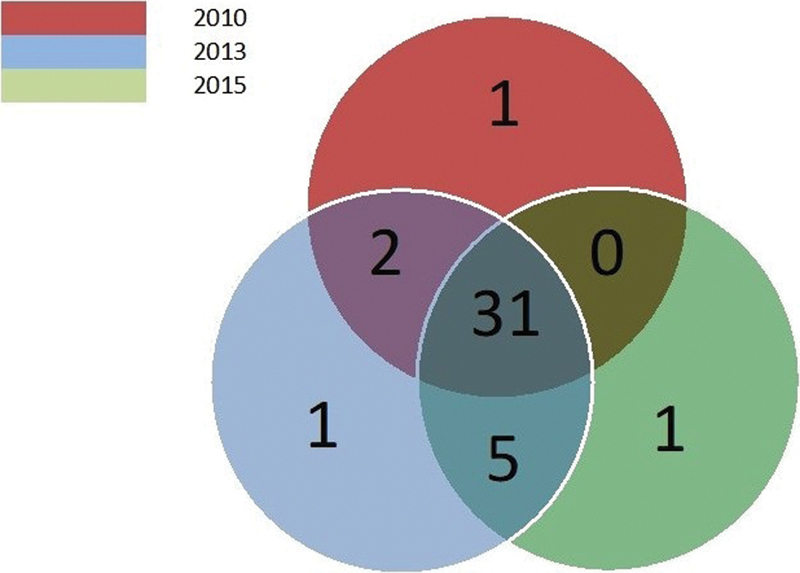
Venn diagram about the variation of gynecology and obstetrics practitioners during the study.

The prescribing physicians did not vary over the 3 periods evaluated (2010, 2013, and 2015), with 76% of the prescribers working in the 3 periods, as presented in Fig. 2.

In the first evaluation period, from July to December 2010, there were 99 cases of acute pyelonephritis during pregnancy. A total of 73 cases (73.7%) received cefuroxime 0.75 g IV three times daily, and one patient received 1.5 g IV three times daily. In 6 cases, cefuroxime was prescribed with an inadequate interval of four times daily (4%). One patient received ampicillin 2 g + sulbactam 1 g IV 3 times daily. A total of 14 cases (14.1%) were prescribed ampicillin 1 g IV, with 1 having an inappropriate interval of 3 times daily. Two patients were prescribed piperacillin/tazobactam 4.5 g IV 3 times daily; 1 patient took ciprofloxacin 0.5 g IV twice daily. In 2 cases, cefuroxime was administered orally 0.5 g twice daily; and in 1 case the oral administration interval was inadequate, being prescribed 3 times daily.

In the second evaluation period, from January to December 2013, there were 116 cases of acute pyelonephritis during pregnancy. A total of 104 patients (89.6%) received a prescription of cefuroxime IV, and in 14 cases the prescription was for twice the usual dose, which was 1.5 g 3 times daily. In 5 cases, (4.3%) cefuroxime IV 0.75 g was prescribed inadequately, twice daily. One patient received piperacillin/tazobactam 4.5 g IV 3 times daily; 1 patient took cefepime 1 g IV every 3 hours; and 5 patients (4.3%) received ampicillin 1 g IV 4 times daily.

In the final evaluation period, from January to December 2015, of the 107 cases admitted with acute pyelonephritis during pregnancy, 103 (96.3%) received cefuroxime IV 3 times daily, with 85 (82.5%) of these given a 0.75 g dosage, and 18 (17.5%) a 1.5 g dosage. Two patients were started on piperacillin/tazobactam 4.5 g IV 3 times daily, and another 2 patients received cefepime 2 g twice daily. This last evaluation period showed no inappropriate choice of antimicrobial, dosage, route, or interval of administration.

**Fig. 3:**
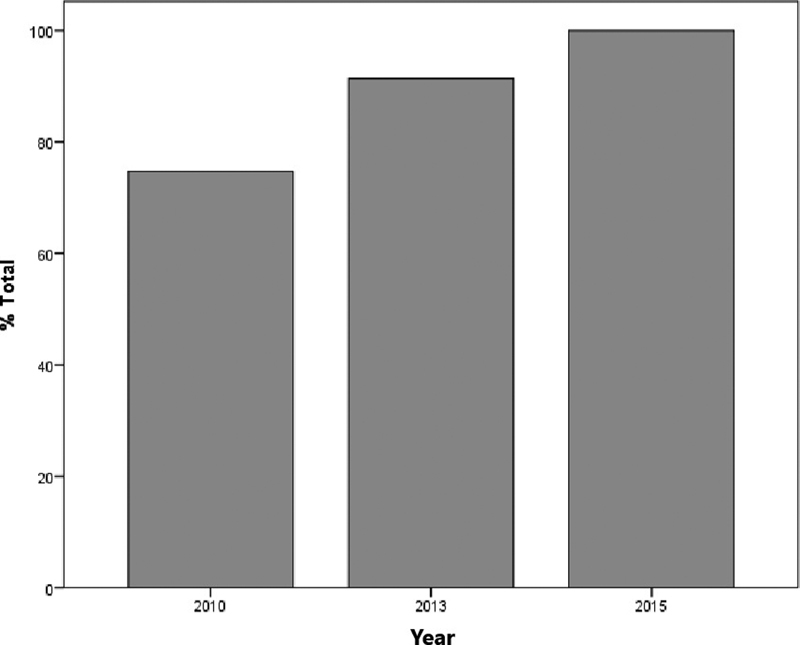
Comparative analysis of appropriate prescriptions in the three periods studied.

The evaluation of the prescribing physicians and of the adequacy of the prescriptions between the different periods studied revealed a significant increase in appropriate conduct for the choice of antimicrobial, route of administration, dosage, and interval, as well as for the total number of interventions following the introduction of the protocol, and again after the implementation of the software application, as shown in Table 1 and in Fig. 3.

**Table 1:** Descriptive table of the “proper conduct” frequency in the prescriptions

	2010	2013	2015	*p*-value
Antimicrobial agent	83.8	95.7	100.0	< 0.001
Dosage	100.0	100.0	100.0	1.000
Route of administration	97.0	100.0	100.0	0.018
Interval between doses	91.9	95.7	100.0	0.004
Completely appropriate prescription	74.7	91.4	100.0	< 0.001

Data presented in percentages (%) and compared by the trend chi-squared test.

## Discussion

The present study described the impact of the inclusion of auxiliary tools on the prescription of antimicrobials by physicians in a maternal-infant hospital, a reference center in the care of pregnant women in the state of Rio Grande do Sul, Brazil. The use of mobile applications in clinical decision making, prescriptions, and for accessing medical information has grown rapidly in recent years, and it is estimated that > 80% of the prescribing professionals have smartphones.^10^ ^11^ ^12^ However, the scarcity of clinically relevant applications on smartphone search platforms and the lack of innovative and original information hinders the dissemination of specialized medical knowledge.^11^ ^12^ ^13^

The development of the protocol in 2011 and the subsequent creation of a smartphone application containing and objectively summarizing the contents of the protocol were achieved, and they could be made available to all physicians at the Hospital Fêmina. In addition, following the 2011 implementation of the treatment protocol for pyelonephritis in pregnancy and the use of the mobile app, a simultaneous and statistically significant increase in the quality of antimicrobial therapy prescription for pregnant women admitted to the hospital with pyelonephritis was observed, from 75% in 2010 to 100% in 2015.

The number of cases included in the present study, the fact that it was conducted in only one hospital, and the possibility that the improvement in prescriptions could have occurred secondarily to the major interest in this subject could be seen as limitations of the present study. Nonetheless, it should be noted that the group of doctors involved in the prescriptions has not varied significantly in the three periods of observation. This is, however, the first study that aimed to evaluate the effect of the use of a specific mobile application on the treatment of infectious diseases during pregnancy, in relation to the quality of prescriptions of antimicrobial agents. Finally, our study has demonstrated that there was a significant improvement in the prescriptions for the treatment of acute pyelonephritis in pregnancy after the inclusion of the protocol and the use of the application.

Smartphone applications that support clinical practice are especially important for physicians as they can provide quick access to information that is consistent with contemporary scientific evidence all over the world.^14^ ^15^

## Conclusion

The present study demonstrates that the design and use of smartphone applications should be encouraged to attain a better quality and accuracy in prescriptions and to include strategies that not only reduce the risk of negative outcomes, but also improve the quality of care and treatment for maintaining the health of both the mother and the baby.
